# Threat expectancies in a VR fear conditioning paradigm follow non-linear extinction patterns but are not influenced by intolerance of uncertainty

**DOI:** 10.1038/s41598-025-23629-0

**Published:** 2025-10-16

**Authors:** Markus Grill, Matthias Kloft, Steffen Anhäuser, Anke Haberkamp

**Affiliations:** 1 Department of Psychology and Psychotherapy, University Witten/Herdecke, Alfred-Herrhausen-Straße 44, 58455 Witten, Germany; 2 Department of Social, Organizational Psychology and Methodology, Philipps Universität Marburg, Marburg, Germany; 3 Department of Mathematics and Computer Science, Philipps Universität Marburg, Marburg, Germany

**Keywords:** Virtual reality, Bayesian analysis, Extinction (learning), Intolerance of uncertainty, Statistics, Human behaviour

## Abstract

In exposure therapy, the degree to which individuals effectively correct their threat expectancies varies considerably. Using appropriate statistical models to capture this variability and investigating potential moderators (i.e. mechanisms of change) of the extinction learning process is therefore important. We hypothesized that a non-linear statistical model would better explain extinction learning trajectories indexed by unconditioned stimulus (US) expectancies than a linear model and that intolerance of uncertainty (IU) might negatively impact extinction. Seventy-one spider-fearful participants completed a two-day realistic virtual reality conditioning paradigm where a spider served as the US. Trial-by-trial US expectancy ratings were collected as the main outcome during acquisition and extinction. We used ordered beta regression in a Bayesian estimation framework to analyze data. As expected, trial-by-trial US expectancy ratings during extinction were substantially better explained by the non-linear ordered beta than a linear statistical model. Non-linear models can therefore more adequately capture interindividual differences in trial-by-trial extinction learning by providing a better fit to such data. However, IU did not moderate US expectancies in our study. Thus, our study supports the notion that US expectancies are largely insensitive to IU.

**Supplementary Information:**

The online version contains supplementary material available at 10.1038/s41598-025-23629-0.

## Introduction

Exposure-based interventions have been shown to be effective for anxiety disorders, obsessive compulsive disorders and post-traumatic stress^1^. The inhibitory learning model of extinction is a contemporary theoretical account explaining the therapeutic mechanism of exposure^2^. It has its roots in Pavlovian fear conditioning, which involves pairing an initially neutral stimulus (conditioned stimulus, CS) with an aversive stimulus (unconditioned stimulus, US), leading to the CS alone eliciting anticipatory fear responses (the conditioned response, CR). This creates a CS-US threat association, and the CS becomes a predictor of threat. The CR can be reduced by extinction learning, where the CS is repeatedly presented in absence of a US. In exposure therapy, this translates to repeated confrontation with fear-provoking stimuli in absence of aversive outcomes.

According to the inhibitory learning model, extinction learning leads to the formation of new CS-no-US associations inhibiting the original threat associations, resulting in reduced fear responses. Craske et al.^2^ also suggest optimization strategies for this process. These include allowing patients to experience a maximum discrepancy between their initial expectation (i.e. expected probability) of threat occurrence (i.e. the US) and the actual rate or frequency of threat occurrence, commonly referred to as expectancy violation. A recent clinical trial focused on changes of threat expectancies during exposure as a predictor of treatment success^3^. In this study, the magnitude of the expectancy violations per se did not predict treatment success. However, what did predict treatment success were aspects that follow the initial expectancy violation. These aspects were patients’ overall change in threat expectancy during the treatment and how well they learned to adjust their threat expectancies after experiencing expectancy violations (i.e. exposure related learning). Especially the exposure related learning showed high inter-individual variability, suggesting that additional factors may influence how well patients adjust their threat expectancies after exposure.

One prominent candidate factor for explaining parts of this variability is Intolerance of Uncertainty (IU), a transdiagnostic psychopathology risk factor defined as the “dispositional incapacity to endure the aversive response triggered by the perceived absence of salient, key, or sufficient information, and sustained by the associated perception of uncertainty”^4^, p. 31. General IU comprises prospective IU (P-IU), related to worry about future events, and inhibitory IU (I-IU), linked to paralysis of cognition or action amidst uncertainty^5^. I-IU may be the specific part of IU that hinders extinction learning by interfering with threat expectancy adaptation through this paralysis of cognition. P-IU, on the other hand, might be less important in this context due to its primary involvement in cognitions about future events. A recent meta-analysis found that high IU (beyond trait anxiety) impairs extinction learning, shown by larger skin conductance responses (SCRs) to the CS+ during threat extinction^6^. Interestingly, another recent review found that exposure therapy also seems to exert an influence on IU levels in individuals: after exposure techniques were applied, reported IU levels decreased compared to pre-exposure^7^. This suggests a potentially bi-directional interaction between IU and extinction learning, highlighting the need to clarify the role of IU not only in objective, but also subjective measures like US expectancies. However, IU’s impact on US expectancies remains unclear, with some studies finding no influence^8^ and others increased expectancies to uncertain threat cues for high IU individuals^9^. Additionally, to our knowledge, whether general IU or I-IU as a subfactor drive the potential impact of IU on threat expectancy adaptation has not yet been specifically investigated.

We propose that the inconsistencies in findings regarding the influence of IU on overt expectancy measure may stem from methodological issues. Specifically, we want to address two key aspects: the use of suboptimal statistical models and experimental paradigms with low external validity. First, accurately representing an individual’s extinction performance is paramount for detecting a moderating influence of any given factor. This requires statistical models that realistically model extinction learning and account well for interindividual differences, whereas suboptimal models can reduce statistical power and hinder moderator detection^10,11^. From a theoretical standpoint, while the update rule of foundational learning models like Rescorla-Wagner^12^ is linear, they generate a characteristic non-linear (i.e. exponentially decaying) learning curve over time. Such a non-linear trajectory has also been suggested by other previous research for extinction learning^13^, potentially rendering linear models not optimally suited when trying to investigate moderating factors such as IU. Thus, identifying the model that best reflects the potentially non-linear trajectory of extinction is a critical first step for a sensitive detection of possible IU influences.

To address the issue of low external validity, we implemented a two-pronged approach. The first relates to typical conditioning paradigms sometimes poorly representing real-life exposure. Hollandt et al.^14^ suggest a considerable methodological gap between typical conditioning paradigms and real-life exposure protocols. They developed a paradigm to better model real-life exposure, which we drew on in the present study. Key optimizations (details in^14^) include: (1) fear consolidation over two days (splitting acquisition/extinction); (2) instructed fear acquisition; (3) pre-trial CS information; (4) return of fear testing; and (5) a medium CS-US reinforcement rate, which allows extinction learning variance and also introduces uncertainty useful for studying IU^15^. Additionally, we aimed to embed this procedure within a maximally immersive context, which can be achieved using virtual reality (VR). VR headsets can display high-fidelity graphics^16^ and thus deliver more realistic, fear-provoking experimental environments while maintaining experimental control^17^. Thereby, VR systems can induce a sense of presence—feeling as if truly being within the virtual environment—prompting more real-life-like behavior^18^. For instance, Mertens et al.^19^ developed a VR differential conditioning paradigm using desk lamp lights as CS and spiders as US. Their paradigm elicited CRs comparable to traditional studies and distinguished between spider-fearful and non-fearful individuals.

In the present study, we combined the paradigms developed by Hollandt et al.^14^ and Mertens et al.^19^ with the aim of maximizing the external validity of our experimental design and to create an environment that enhances our ability to detect potential moderating effects of IU on US expectancies in extinction learning. In our VR environment, two different light colors of a desk lamp served as the CS and 3D-animated spiders served as the US. Our study sample consisted of individuals with a high fear of spiders, to ensure appropriate aversive responses to the US. Note that spider-fearful individuals displayed IU scores in previous research which are elevated compared to non-fearful controls, but also varied between low IU and high IU scores^20^.

Taken together, the main goals of our study were to determine whether our non-linear model provides a better fit to the trajectory of US expectancy ratings in extinction than a linear model and if higher IU (or I-IU, over and above trait anxiety) negatively impacts inhibitory learning during extinction in the domain of US expectancy ratings. We formulated the following hypotheses:

*H0.1 Acquisition of CS-US associations (manipulation check):* We expect that participants successfully learn to associate the CS+, and not CS−, with the US. Therefore, we expect participants’ US expectancy ratings in response to the CS+ to increase across trials, while US expectancy ratings in response to the CS− should remain near zero.

*H0.2 Valence*,* fear*,* and disgust of CS+/− and the spider (manipulation check):* CS + should have more negative valence and higher fear/disgust ratings than CS− and individuals should report negative valence, fear, and disgust in response to the spider US at the end of the acquisition.

*H1 Assessment of non-linear regression model fit*: We expect US expectancy to decrease in a non-linear manner across trials during extinction. The decrease is expected to roughly follow a logistic curve rather than a straight linear trend. Consequently, a non-linear model tailored to this type of response pattern should fit the data better than a linear model.

*H2.1 Effect of IU on extinction:* We expect high IU individuals to have poorer extinction learning than low IU individuals, resulting in differences in the shape of the curve between individuals. High IU Individuals are expected to require more trials to reduce their US expectancies to the same extent as low IU individuals. On average, we expect all individuals to show successful extinction at the end of the extinction phase (indexed by a significant decrease in US expectancies across extinction).

*H2.2 Effect of I-IU on extinction:* We believe that I-IU may be the specific component of general IU that prevents high IU individuals from appropriately updating threat expectancies. As the I-IU and P-IU subscales have also been criticized in the literature^6,21^, we intend to investigate this aspect in an exploratory manner.

*H2.3 Effect of IU on US expectancy in extinction controlling for trait anxiety:* Similar to the results shown in^6^ for SCRs, we also expect IU to be specifically associated with extinction over and above trait anxiety.

## Results

### Acquisition of CS-US associations

We used the ordered beta regression model to conduct the manipulation check (H0.1), predicting the US expectancy ratings with the fixed effects *CS-Type*, *Trial* and their interaction. We also allowed for a random intercept and slope for Trial per participant (*ID* in the pseudo-code), and a random intercept per Trial, resulting in the following pseudo-code for the predictive term of the model:

1 $$ER~\sim 1+Trial+CS\,Type+Trial \times CS\,Type+(1+Trial|ID)+(1|Trial).$$

**Fig. 1:**
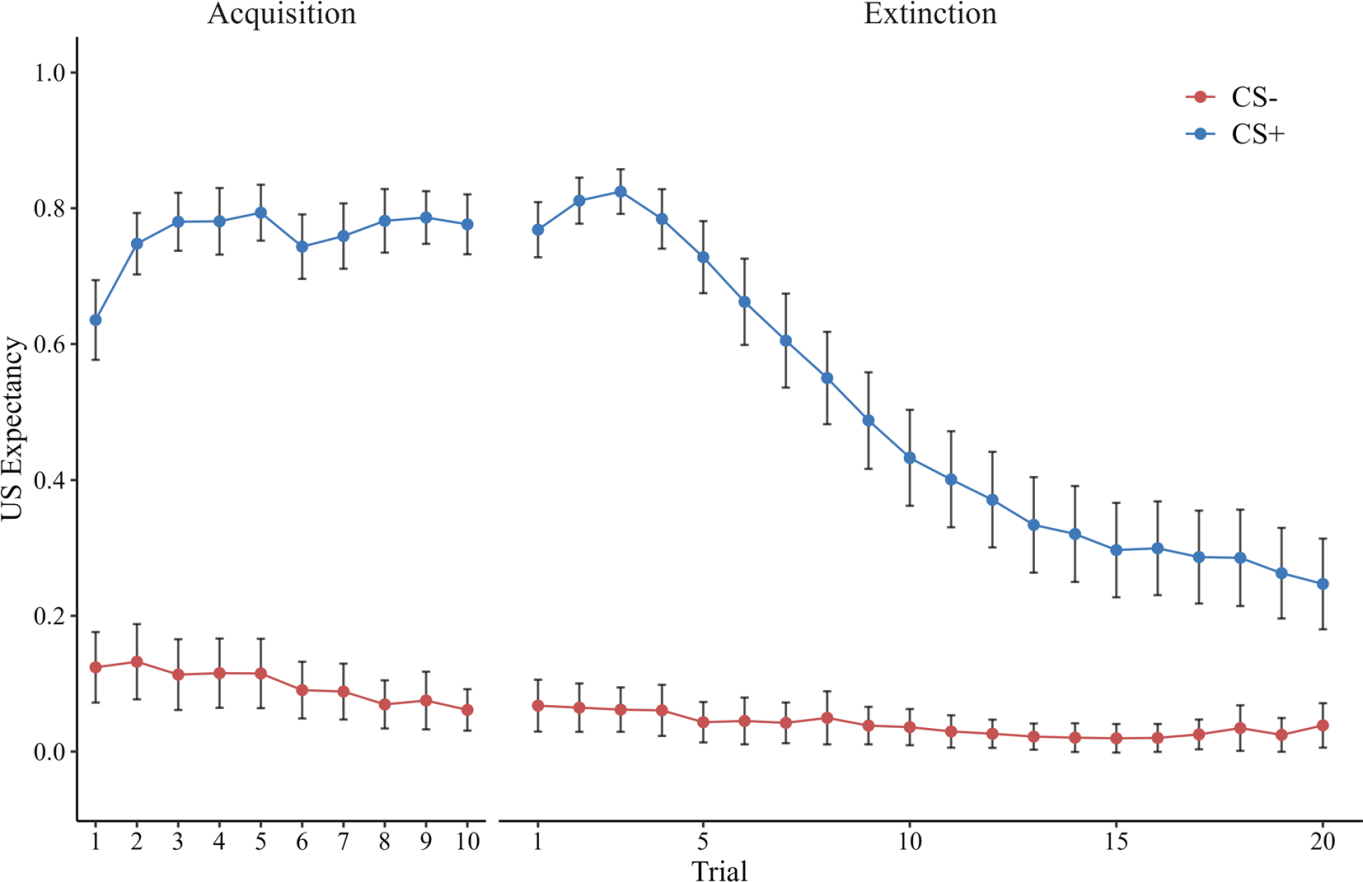
Mean trial-by-trial US expectancy ratings in acquisition and extinction for CS + and CS−. Error bars indicate 95% CIs.

We coded *CS-Type* with effects coding (CS− = −0.5, CS + = 0.5) to test whether the stimulus conditions had the intended effect (i.e., a positive difference between conditions). As expected, the model indicated that participants in our study robustly acquired the association of the CS+ and the US, indicated by significant regression coefficients for *CS-Type* (logit scale, *b*_*CS−Type*_ = 2.56, 95% CI [2.33, ∞]) and *Trial×CS-Type* interaction (logit scale, *b*_*Trial×CS−Type*_ = 0.10, 95% CI [0.05, ∞]). The table with all parameter estimates for the manipulation check can be found in the online supplement (see Table S2). See Fig. 1 for a graphical illustration of US expectancy trajectories in acquisition and extinction. Individuals reported markedly increased subjective fear and disgust, as well as more negative valence in response to the CS+ (fear: *M* = 6.46, *SD* = 3.00; disgust: *M* = 4.83, *SD* = 3.26; valence: *M* = 6.33, *SD* = 3.00) compared to the CS− (fear: *M* = 1.51, *SD* = 2.12; disgust: *M* = 1.01, *SD* = 1.86; valence: *M* = 2.09, *SD* = 2.21) at the end of acquisition. These differences were all significant at the level of α = 5% (fear: *t*(69) = 13.00, *p* < 0.001, Cohen’s *d* = 1.54, 95% CI [1.20, 1.89], disgust: *t*(69) = 9.04, *p* < 0.001, Cohen’s *d* = 1.07, 95% CI [0.78, 1.36], valence: *t*(69) = 10.28, *p* < 0.001, Cohen’s *d* = 1.22, 95% CI [0.91, 1.52]), suggesting successful fear acquisition. Descriptively, participants’ response to the spider were all towards the upper (i.e. more negative) ends of the scales, which went from 0 as the lowest possible rating to 10 as the highest one (fear: *M* = 7.61, *SD* = 2.39; disgust: *M* = 7.85, *SD* = 2.45, valence: *M* = 8.15, *SD* = 2.10). Thus, the spider seemed to elicit sufficient aversive responding which was then successfully coupled with the CS + during acquisition.

### Modeling foundations for the analysis of fear extinction data

We based our analyses for H1 (model fit comparison of linear regression and non-linear ordered beta regression) and H2 (H2.1: effect of IU on US expectancy in extinction, H2.2: effect of I-IU on US expectancy in extinction, H2.3: effect of IU on US expectancy controlling for trait anxiety) on the following baseline model:

2 $$ER~\left( {CS+} \right)~\sim 1+Trial+(1+Trial|ID)+(1|Trial)$$

As we were mainly interested in participant responses to the CS+, we calculated our models including only CS + trials (i.e. removing the CS− Type as a predictor). For the linear regression model (H1), the pseudo code in Eq. (2) refers to the mean of the normal distribution while for the ordered beta model, it refers to ${{{\varvec{\upzeta}}}_{it}}$ (see Eqs. 4 and 5). The parameter estimates and detailed descriptions of the models concerned in the analyses of H1, H2.1 and H2.3 can be found in Table 1. Models including the US expectancy ratings of the CS− trials as the outcome were calculated as controls and can be viewed in the online supplement (see Table S3).

**Table 1:** Details for different models predicting US expectancies in extinction in response to the CS+.

Parameter	Linear baseline	Ordered beta baseline	Ordered beta IU	Ordered beta I-IU
	Est. [95% CI]	Est. [95% CI]^a^	Est. [95% CI]^a^	Est. [95% CI]^a^
*Fixed effects*				
Int.	**0.81** [0.74; 0.88]	**1.88** [1.56; 2.19]	**1.89** [1.57; 2.20]	**1.87** [1.56; 2.19]
γ	n.a.	**2.35** [2.27; 2.44]	**2.35** [2.27; 2.44]	**2.35** [2.27; 2.44]
b_Trial_	− **0.03** [− 0.04; − 0.03]	− **0.24** [− 0.28; − 0.19]	− **0.24** [− 0.28; − 0.19]	− **0.23** [− 0.28; − 0.19]
b_IU/I−IU_	n.a.	n.a.	− 0.06 [− 0.33; 0.19]	− 0.11 [− 0.37; 0.14]
b_Trial×IU/I−IU_	n.a.	n.a.	0.01 [− 0.04; 0.05]	0.02 [− 0.03; 0.06]
*Random effects*				
ID: Intercept	**0.16** [0.14; 0.20]	**1.04** [0.86; 1.26]	**1.05** [0.87; 1.28]	**1.05** [0.87; 1.27]
ID: *SD*(b_Trial_)	**0.02** [0.01; 0.02]	**0.19** [0.15; 0.23]	**0.19** [0.16; 0.23]	**0.19** [0.16; 0.23]
ID: Cor(Int.,b_Trial_)	− 0.14 [− 0.39; 0.12]	− **0.49** [− 0.66; − 0.27]	− **0.49** [− 0.66; − 0.28]	− **0.49** [− 0.66; − 0.28]
Trial: Intercept	**0.06** [0.04; 0.09]	**0.20** [0.13; 0.3]	**0.20** [0.13; 0.3]	**0.20** [0.13; 0.29]
Model fit	Est. [95% CI]^b^	Est. [95% CI]^b^	Est. [95% CI]^b^	Est. [95% CI]^b^
*R²* conditional	0.84 [0.83; 0.85]	0.88 [0.87; 0.88]	0.88 [0.87; 0.88]	0.88 [0.87; 0.88]
*R²* marginal	0.35 [0.27; 0.43]	0.49 [0.44, 0.53]	0.49 [0.43, 0.53]	0.49 [0.43, 0.53]

These models included only the course of US expectancies in extinction, and only in response to the CS+. Baseline models did not include any IU scores as predictors. The IU model and I-IU models included the general IU and I-IU scores respectively. The γ parameter is specific to ordered beta regression and therefore not present in the linear model. In the random effects section, ID denotes random effects per participant, and Trial denotes the random effect per trial. “*R²* conditional” is based on random and fixed effects estimated in the models. “*R²* marginal” is based only on the estimates for the fixed effects. Model parameters in bold indicate estimates where the 95% CI did not include zero. Cor = correlation. ^a^One-sided lower and upper 95% CIs. ^b^ Two-sided 95% Cis.

### Linear vs. non-linear learning patterns during extinction

We calculated the baseline model in Eq. (2) as a standard mixed effects linear regression (i.e., assuming a normal distribution of ratings) as well as a non-linear ordered beta regression to analyze if the non-linear model provides a better fit than a linear model to the US expectancies in extinction. The models were compared by means of *R*^2^ and graphical posterior predictive checks^22^.

The difference in explained variance was 4% (linear: *R*^2^ = 0.838, 95% CI [0.830, 0.845]; non-linear: *R*^2^ = 0.876, 95% CI [0.870, 0.883]) when including the fixed effects (the actual predictors we were interested in) and the random effects (trial and person specific residuals). Importantly, when only considering the fixed effects, the difference was much larger with 14% (linear: *R*^2^ = 0.353, 95% CI [0.266, 0.428]; non-linear: *R*^2^ = 0.494, 95% CI [0.443, 0.533]). Moreover, we computed the Bayes factor for the linear vs. the non-linear model. The Bayes factor > 5000 in favor of the ordered beta model also suggested extreme evidence^23^ for a non-linear trajectory of US expectancies.

**Fig. 2:**
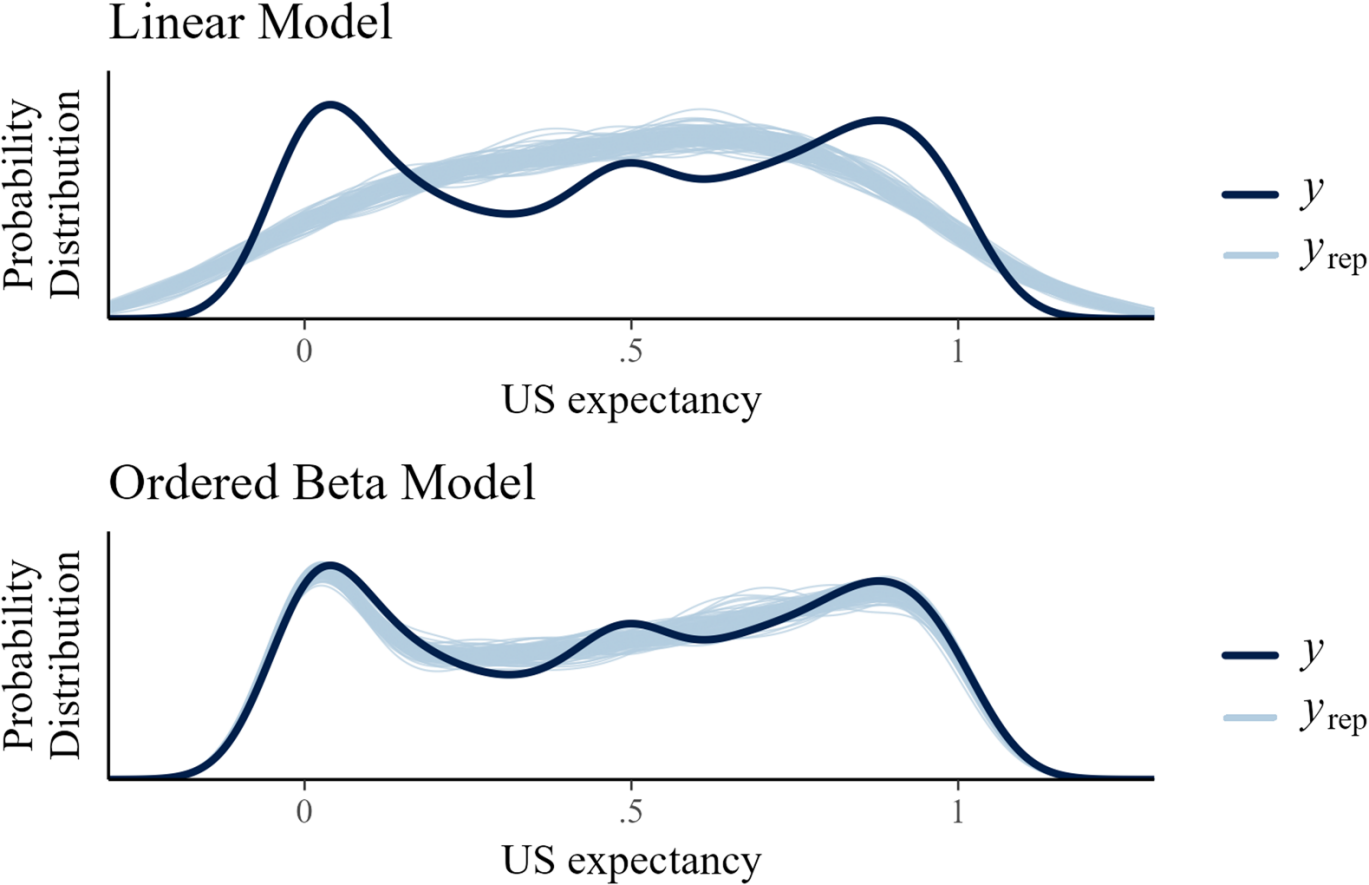
Posterior predictive plots. Overlays of the marginal distributions of empirical expectancy ratings (dark blue) and predicted expectancy ratings by the linear and non-linear model (light blue).

Providing graphical posterior predictive checks, Fig. 2 shows overlays of the marginal distributions of empirical (dark blue line) and predicted (light blue line) expectancies by the linear and non-linear model. Predicted ratings stem from 100 random draws of each model’s posterior samples. The linear model (top panel) overpredicts ratings in the middle range while it underpredicts ratings at the ends of the scale. Further, the linear model predicts ratings outside of the possible range of [0, 1] (the extent to which the empirical ratings lie out of this range is due to the smoothing applied by the plotting function). In contrast, the predictions of the ordered beta model (bottom panel) more closely mimic the distribution of empirical ratings, except for the distributional mode and valley in the middle.

Combining the evidence of the explained variance, the model comparison Bayes factor and the graphical posterior predictive checks, we conclude that, in line with our expectations, the non-linear ordered beta model described the overall trajectory of the US expectancy ratings during extinction better than the linear model.

### Influence of IU and I-IU on US expectancies in extinction learning

In our analysis of H2.1 (influence of IU on US expectancies in extinction), we added the IU scores and their interaction with *Trial* as fixed effects to our baseline model in Eq. 2. Thereby we assessed whether IU explained variance in the trajectory of the US expectancy ratings across the extinction trials (Trial×IU):

3 $$US\,Expectancy\,\left( {CS+} \right) \sim 1+Trial+IU+Trial \times IU+(1+Trial|ID)+(1|Trial)$$

Details for the parameter estimates can be found in Table 1. The effect of IU on the US expectancies trajectory during extinction (i.e. the *Trial×IU* interaction) was not significant with *b*_*Trial*×*IU*_ = 0.01 (logit scale, 95% CI [− 0.04, ∞]).

We also compared this model to the baseline model in Eq. (2) by means of ELPD_loo_ (leave-one-out expected log pointwise predictive density^24^; higher ELPD_loo_ values indicate better model fit) to assess whether the model containing IU had the better fit. The difference in ELPD_loo_ was − 0.04 (*SE* = 0.5) in favor of the baseline model, which, in relation to the size of the standard error is not a meaningful difference. We further computed the Bayes factor for the model comparison, which was *BF*_01_ = 1733 in favor of the baseline model. Also, the amount of explained variance (*R*^2^ = 0.491, 95% CI [0.436, 0.530], considering only fixed effects, did not change substantially compared to the baseline model (*R*^2^ = 0.494, 95% CI [0.443, 0.533]).

Against our expectations, all model comparisons indicated that the model including IU provided a worse fit than the baseline model, probably due to the additional uncertainty introduced by the additional parameters.

In our analyses for H2.2 (influence of I-IU on US expectancies in extinction) we repeated the analyses we conducted for H2.1 but substituted IU scores with I-IU scores. Note that we did not have specific expectations regarding the results of these analyses. The effect of I-IU on the trajectory of the extinction (i.e., the interaction between *Trial* and *I-IU*) was not significant with *b*_*Trial×I−IU*_ = 0.02 (on a logit scale, 95% CI [− 0.03, ∞]). Similar to the previous model, the difference in ELPD_loo_ was − 0.06 (*SE* = 1.0) in favor of the baseline model. Further, we computed a Bayes factor of *BF*_01_ = 1253 in favor of the baseline model. Also, the explained variance was less than for the other models with *R*^2^ = 0.489 (95% CI [0.434, 0.532], considering only fixed effects). We conclude that I-IU, similar to general IU scores, did not provide a better fit to the data than the baseline model in Eq. (2).

Since both IU and I-IU did not improve the model fit of our non-linear beta regression model, calculating the model with the STAI-T scores to assess the specificity of IU-related effects as described in H2.3 would not provide any meaningful insights. Therefore, we do not report these results here. We still calculated the models, but, as expected due to the null-effects of the models calculated for H2.1 and H2.2, the IU scores had no incremental predictive value over the STAI-T scores (see Table S4 in the online supplement for details). Additionally, in an exploratory analysis, we calculated an ordered beta model with SPQ scores as the moderator variable to investigate the possibility that spider fear severity was the main influential factor on US expectancies, potentially obscuring effects of IU or I-IU. However, the effect of SPQ scores on extinction trajectories was not significant *b*_*Trial×SPQ*_ = 0.01 (on a logit scale, 95% CI [− 0.01, 0.02]), suggesting that spider fear levels did not obscure the effects of IU or I-IU on extinction learning in our study (see the full table with model parameters in Table S7 in the online supplement).

## Discussion

This study investigated whether non-linear models better describe US expectancies than linear models during fear extinction in a VR conditioning paradigm, and whether high Intolerance of Uncertainty (IU) or its sub-factor I-IU predicts poorer extinction learning. Our experimental paradigm adapted methods from Hollandt et al.^14^ and Mertens et al.^19^ to increase external validity, using a VR setting with 3D-animated spiders as the US. The non-linear model provided a significantly better fit to the data. However, contrary to our hypotheses, we found no significant relationship between IU or I-IU and the extinction learning trajectory.

The superior fit of the non-linear model is consistent with previous research showing that learning can be non-linear^13^. This pattern is intuitively plausible: in the extinction phase, an individual is initially uncertain if the US absence indicates safety or is just an anomaly. Only after sufficient evidence, confidence increases and learning accelerates, marked by then rapid decreases in US expectancies. Supporting this notion, a review found that people suffering from anxiety disorders display difficulties in adapting their learning rates to changing environments, i.e. learning to interpret a former threat signal as now safe^25^. This process mirrors real-life exposure therapy, where a patient’s high threat expectancies (e.g. “I am sure that I will suffer a heart attack if I run up these stairs”) may only drop significantly after several sessions establish a new sense of safety. The amount of evidence needed might be disproportionately high in anxiety patients, explaining an initial plateau in their extinction learning. However, such an initial plateau has not necessarily been observed in previous studies. Gromer et al.^26^, for example, found an instant decline of US expectancies instead of an initial plateau. This may potentially stem from different experimental settings (e.g. instructed vs. uninstructed conditioning or the US used). In our study, the US had a pre-existing negative emotional valence for subjects participating (as in real-life exposure). This potentially resulted in harder-to-unlearn US expectancies as opposed to e.g. an electrical shock, which might be universally perceived as unpleasant but does not necessarily hold pre-existing biographical significance. We believe that this further supports our notion of creating more externally valid experimental paradigms aiming to mimic real-life scenarios as closely as possible.

While linear models remain useful, our data suggests that in scenarios similar to ours, non-linear models can provide a more accurate representation of reality, increasing statistical power to detect effects that might otherwise be missed^10^. The model choice is critical, as inappropriate statistical models can substantially alter effect sizes and statistical significance in fear conditioning studies^27^. The same authors also found that mixed-effects models are generally underutilized compared to ANOVAs or t-tests. Since fear extinction is susceptible to interindividual differences, datasets may contain high variability^28^. Mixed-effects models are better suited to handle this variability by incorporating elaborate random effects structures^29^, potentially making them more appropriate for analyzing trial-by-trial fear conditioning data and understanding extinction mechanisms.

Given the high interindividual variability in exposure-related learning^3^, another important research avenue for improving exposure therapies is identifying factors that moderate their effectiveness. We investigated IU as a potential factor negatively influencing exposure-related learning due to its robust associations with poor extinction learning in SCRs^6^. By combining a more realistic laboratory setting with an appropriate statistical model, our study was designed to eliminate potential methodological reasons for past studies failing to find meaningful effects of IU on overt measures, such as US expectancies.

Contrary to our hypotheses, however, we did not find any meaningful associations of IU (or I-IU) with US expectancies. This result aligns with other recent research^8,30^, leading to our conclusion that US expectancies may be largely insensitive to IU. This raises the question which, if an effect truly exists, measures adequately capture IU’s influence in conditioning contexts. Possibly, our assumption that the effect of IU on US expectancies is similar in size than that of IU on psychophysiological measures may have been suboptimal and the true effect of IU on US expectancies might be smaller. If that was the case, the issue would become one of statistical power, as our study was not powered to detect effects smaller than those in the context of IU influences on psychophysiological measures. Given the effect sizes in our sample, we believe that to be unlikely. Another important aspect here is the format of the response scales. For example, two studies found that high IU individuals reported higher fear levels during fear acquisition in response to the CS+^31,32^. Both of these studies used response scales from 0 to 100, allowing for bigger variances in responding. This may have led to a higher sensitivity for detecting effects of IU on an overt measure, which scales e.g. from 0 to 10 may not allow. However, since we also used a scale of 0 to 100 for our expectancy ratings, this most likely was not an issue in our study. More, we conclude that the lack of an association between IU scores and US expectancies is also not due to limited variance in the IU scores of our sample, since our results here are comparable with results commonly found using the IUS-12 to measure IU (see the supplementary material of Morriss et al.^6^.

A compelling alternative to the aforementioned points is overt avoidance behavior. For instance, Flores et al.^33^ linked IU to a direct display of avoidance where healthy individuals high in P-IU (but not I-IU, which we hypothesized in our study) showed more avoidance behavior than those low in P-IU in a conditioning paradigm. Another study also using a fear conditioning paradigm found that high-IU individuals showed more avoidance of not only the threatening CS + but also similar, safe stimuli^34^. The evidence is not definitive, however, as other studies did not find this association with avoidance^35,36^. These conflicting results may be e.g. due to differences in paradigm-induced uncertainty, warranting further investigation (see^15^ for a more detailed review).

Nevertheless, integrating our results with those of Hunt et al. and Flores et al.^33,34^, IU may influence US expectancies in extinction via avoidance rather than directly. If high-IU individuals avoid fear-inducing stimuli, the likelihood of them experiencing an expectancy violation is reduced to zero. This prohibits inhibitory CS-US associations from forming, maintaining anxiety^37^. While this seems to be a potentially important mechanism for how anxiety is maintained in high IU individuals, it does not contribute to explaining the high interindividual variability in exposure related learning found by Pittig et al.^3^. Our data overall suggest that IU does not play a critical role here. Thus, future research should further aim to clarify how IU might influence more overt extinction learning processes.

### Limitations

The present study also has limitations that need to be considered. First, our sample consisted of predominantly female participants. Given the gender distribution of spider fear in the general population^38^, this is not surprising. Combined with the limited age distribution in our sample, this still limits the generalizability of findings. Relatedly, we also decided to recruit highly spider-fearful rather than spider-phobic individuals. Although spider-fearful individuals behave similarly to spider-phobic individuals^39^, a spider-phobic sample might still respond somewhat differently than ours.

Second, we did not measure psychophysiological responses such as SCRs (as we explicitly designed our study to focus on US expectancies). SCRs could potentially also follow a non-linear trajectory during extinction. Such a finding would support the idea of using non-linear modeling in this domain as well. Measuring extinction learning in different domains provides a more complete picture of the processes at work in any given sample. Given the complexity of IU influences on extinction processes^15^, the use of non-linear models in general may lead to a more refined understanding of the effects of IU on these processes.

Third, it may be considered arbitrary to predict spider appearances with differently colored lights. Our virtual environment may therefore have appeared less plausible, which may have had a negative impact on participants’ sense of presence^40^. The virtual environment could be designed to include more realistic predictors of spiders, such as the presence of spider webs in a room that participants have to enter. Such environments may be more susceptible to confounding influences due to their complexity. Nevertheless, we believe that it would be valuable to further improve the external validity of our study design by taking such steps and may result in participant more closely resembling their real-life behavior^40^.

Finally, we chose to measure IU with the commonly used IUS-12. This is not necessarily a limitation per se, as the psychometric quality of this measure is adequate^41,42^. However, there is another measure called the Intolerance of Uncertainty Index (IUI^43^. The IUI was developed to better measure behavioral manifestations of IU in common anxiety symptoms. As we were investigating the possibility of high IU manifesting in anxiety disorders via potential influences on US expectancies, this may have been the more appropriate measurement. Unfortunately, the IUI was not available in German at the time of the study. Also, we used the STAI-T as a measure of trait anxiety, it is important to note that this scale may capture a broader construct of negative affectivity rather than trait anxiety specifically^44^. This potential lack of specificity should be generally considered when evaluating results, although we believe it to be a non-essential aspect in our study since we did not conduct our confirmatory analyses of hypotheses including STAI-T scores due to a lack of association between IU scores and US expectancies.

### Conclusion

The present study investigated US expectancy trajectories during extinction learning and whether these are influenced by IU. We used a VR conditioning paradigm, particularly focusing on optimizing the external validity of our study. Trial-by-trial trajectories of US expectancies in extinction could be better explained by a non-linear than a linear statistical model. This shows that extinction appears to occur non-linearly, such that individuals need a few extinction trials before they “trust” their new experiences of safety, and inhibitory learning shows its effect, before the learning rate decreases again and effectively “flattens out” as the US expectancies approach a stable, asymptotic floor. Concluding from our results, non-linear statistical models appear to be more appropriate when modeling trial-by-trial extinction learning indexed by US expectancies. Contrary to our hypothesis, IU did not show a moderating effect on US expectancies. Possibly, IU exerts its influence on US expectancies not directly, but indirectly by increasing the avoidance behavior of high IU individuals^33,34^, which prevents them from forming new inhibitory CS-safety associations^37^. This issue should be addressed in future research. Finally, this study has further demonstrated how VR can be used in conditioning paradigms to create more externally valid experimental settings, potentially leading to more realistic participant behavior in the laboratory.

## Methods

The present study was preregistered on the Open Science Framework (OSF, https://osf.io/6gjyp). Data and analysis code are also accessible on OSF (https://osf.io/2nqv4/). The study was approved by the Ethical Committee of the Faculty of Psychology (Philipps Universität Marburg, case number 2020-53k), and carried out in accordance with the provisions of the World Medical Association Declaration of Helsinki.

### Participants

We refer to participants in our study as “spider-fearful” instead of “spider-phobic” as we did not assess whether they fulfilled all criteria of clinical spider phobia^45^. Importantly, previous research has shown that individuals not meeting all those criteria exhibit similar reactions to spiders as individuals who do^39^.

We determined the sample size required for our study by conducting a power analysis via data simulation in R^46^. We aimed for a power of 0.80 to detect the effect of *r* = 0.35 for influence of IU on the expectancy trajectories in extinction. This is in line with the effect sizes of IU on extinction learning in previous studies in the domain of SCRs, and we assumed a similar effect size in our study^6^. Details of the rationale for our power analysis can be found on OSF (https://osf.io/2nqv4/). The power analysis revealed that our sample should consist of *N* = 70 individuals to detect our desired minimum effect size.

We advertised our study via email announcements, the online research participation platform Sona (https://www.sona-systems.com), flyers, and word-of-mouth at the universities of Marburg and Gießen. Fear of spiders was assessed using the Spider Phobia Questionnaire (SPQ), with individuals scoring above the 75th percentile of the gender norm sample considered to be highly fearful of spiders (scores of 10 and above for males and 14 and above for females, see^47^). Individuals were excluded if they scored below the 75th percentile on the SPQ, were receiving psychotherapeutic/psychopharmacological treatment, or had elevated depressive symptoms (indicated by BDI-II scores above 18^48^). Additionally, we also planned to exclude participants who did not learn the CS-US contingencies during acquisition (assessed by checking the mean differences between US expectancies in response to the CS + and CS− after acquisition). Importantly, no participant had to be excluded due to not learning the CS-US contingencies.

All participants gave written informed consent and received either course credit or monetary compensation. A total of 300 individuals were screened for eligibility in an online screening. Of those, 81 withdrew prior to screening completion, 37 did not meet the spider fear criterion, 20 reported elevated depressive symptoms, and nine received psychotherapeutic / psychopharmacological treatment. Of the remaining 153 potentially eligible participants, 47 did not book an appointment at the laboratory, 29 did not attend their booked appointment, five abandoned the experiment during acquisition, and the data of one participant was removed from our sample due to a technical error, resulting in a final sample size of *N* = 71. Demographic information and questionnaire scores for our sample can be found in Table 2.

**Table 2:** Sample characteristics (N = 71).

Demographics			
Mean age in years (SD)	23.96 (6.91)	Geographic background	
Sex		European	67
Female	56	Asian	4
Male	15	Ethnicity	
Education		White	67
Pupil	2	Black	1
Secondary school certificate	1	BIPoC	3
Vocational diploma	1		
High school	44		
Academic degree	23		

Mean questionnaire scores (SD)			
SPQ	18.82 (3.59)	IUS-12	29.70 (7.28)
FSQ	62.73 (19.54)	IUS-12 I	13.46 (3.82)
BDI-II	4.94 (4.32)	IUS-12 P	16.24 (4.44)
STAI-T	41.00 (8.05)		

SPQ = Spider Phobia Questionnaire, FSQ = Fear of Spiders Questionnaire, BDI-II = Becks Depression Inventory II, STAI-T = State Trait Anxiety Inventory – Trait Version, IUS-12 / I / P = Intolerance of Uncertainty Scale – 12 / Inhibitory Subscale / Prospective Subscale, BIPoC = Black / Indigenous / Person of Color.

### Materials

#### Self-report measures

We measured spider fear with the SPQ (German version by Hamm^47^; original by Klorman et al.^49^, and corroborated the findings of the SPQ with the Fear of Spiders Questionnaire (FSQ; German version by Rinck et al.^50^; original by Szymanski and O’Donohue^51^). The SPQ is a 31-item self-report scale measuring the severity of spider phobia symptoms using a dichotomous response format (“Yes” vs. “No"). It has been shown to be reliable and valid^47^. Cronbach’s α as an indicator of internal consistency was .63 in our sample. The FSQ is an 18-item self-report scale that also measures severity of spider phobia symptoms. Items are rated on a 7-point Likert-type scale from 0 = *not at all* to 6 = *very much*. It is frequently used to corroborate findings from the SPQ (e.g. ^20,52^) and has shown good psychometric qualities^50^. McDonald’s ω_t_ as an indicator of internal consistency was .94 and McDonald’s ω_h_ as an indicator of g-factor saturation was .68 in our sample. Depressive symptoms were assessed using the Beck-Depression-Inventory-II (BDI-II; German version by Hautzinger et al.^48^, original by Beck et al.^53^). The BDI-II is a 21-item self-report scale measuring the severity of depressive symptoms using a response scale from 0 to 3. It has demonstrated excellent reliability and validity^48^. McDonald’s ω_t_ was 0.82 and McDonald’s ω_h_ was 0.67 in our sample. We measured IU with the Intolerance-of-Uncertainty-Scale Short Form (IUS-12; German version by Dietmaier et al.^42^, original by Carleton et al.^41^). The IUS-12 measures general IU using 12 items on a 5-point Likert-type scale from 0 = *not at all characteristic of me* to 4 = *entirely characteristic of me.* It provides a general IU scale and two subscales I-IU and P-IU, consisting of six items each. Its psychometrics have been shown to be adequate^42^. The internal consistency in our sample was ω_t_ = 0.87 for general, ω_t_ = 0.82 for inhibitory, and ω_t_ = 0.83 for prospective IU. McDonald’s ω_h_ was 0.57, 0.65, and 0.60 respectively. Trait anxiety was measured via the State-Trait-Anxiety-Inventory-Trait (STAI-T; German version by Laux et al.^54^; original by Spielberger^55^). The STAI-T is a 20-item scale measuring trait anxiety. Responses are given on a 4-point Likert-type scale from 0 = *not at all* to 3 = *very much*. Psychometric qualities of the scale are good^54^ and the internal consistency in our sample was ω_t_ = 0.89 while g-factor saturation was ω_h_ = 0.63. Although intended to measure trait anxiety, recent results suggest that STAI-T may not be a specific measure of trait anxiety per se, but rather a broader measure of general negative affectivity^44^.

#### VR environment

**Fig. 3:**
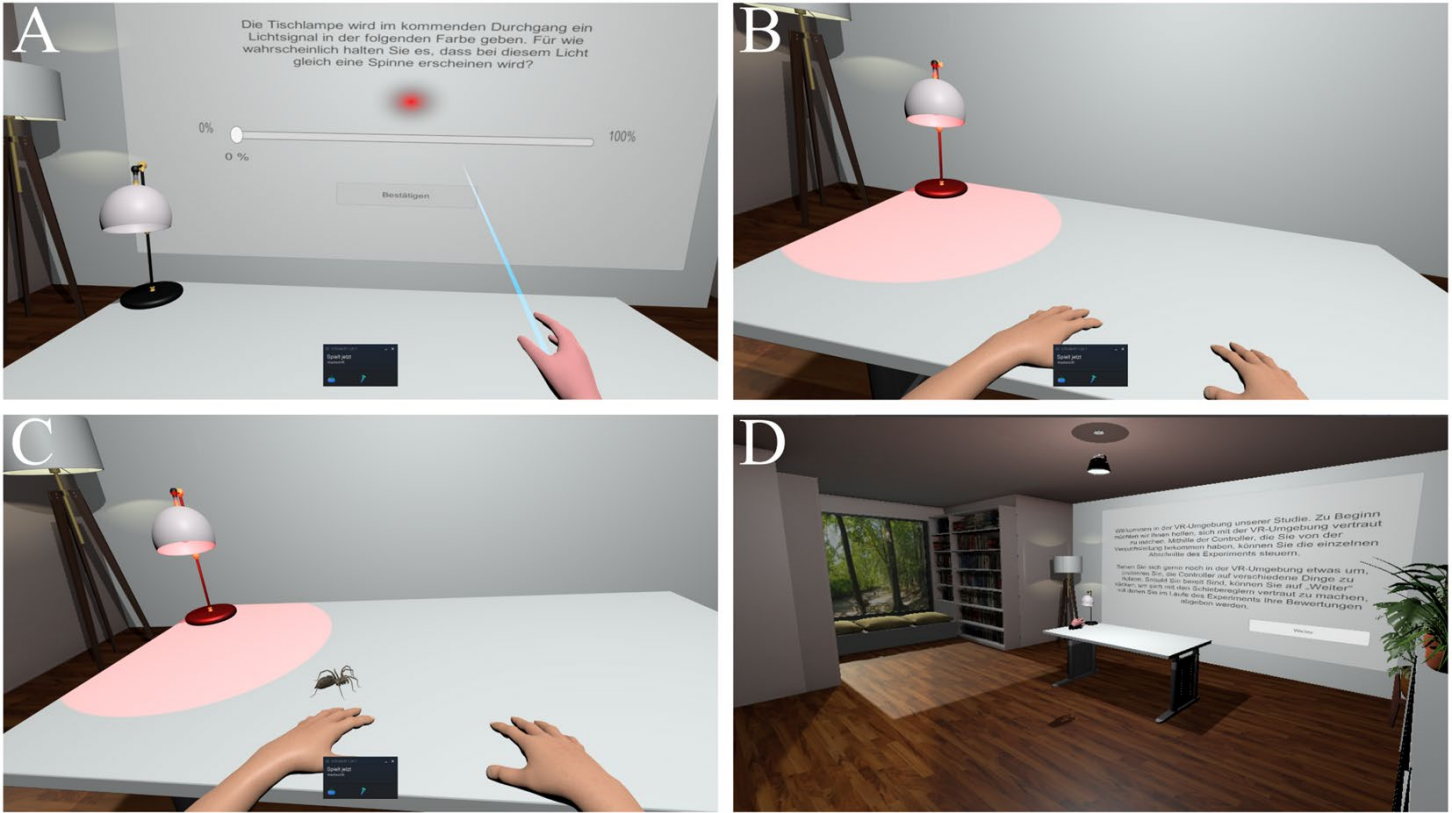
Screenshots of the VR environment. **A** Depiction of the visual analogue rating scale presented prior to each CS presentation per trial. **B** Depiction of the light signal CS. **C** Sample picture of a reinforced CS + trial with the spider present. **D** Depiction of the VR environment from an overview perspective. Note that the small window in the bottom of screenshots **A**–**C** was not visible within the VR environment.

We based our virtual environment on Mertens et al.^19^, developed it in the Unity Game Engine (version 2021.3.26f1), and used the PICO Neo 3 Pro (Pico, China) for its presentation. The field of view was set to 60° to facilitate focus on the table and desk lamp. In the virtual room, participants were seated at a table in a first-person perspective. They used either a right- or left-hand controller (depending on handedness) to interact with the environment. The controller was only visible in the virtual environment when interactions were possible, otherwise two arms resting on the table were displayed. The desk lamp producing the blue / red light signal (CS) was placed on the table, and the wall opposite of participants display instructions and rating scales. A 3D model of a wolf spider (*Lycosidae*) with pronounced abdomen and chelicerae^56^ was used as the US. The spider always appeared by crawling over the table’s edge, disappeared by fading out, and could move along six different paths (see Fig. S1 in the online supplement for an illustration of the paths). The spider’s movement speed varied throughout each path, and the spider was designed to move as unpredictably as possible to increase fear and disgust responses^20^. In a small pilot study (*n* = 4), we collected feedback on the overall look and feel of the VR environment, as well as fear and disgust ratings of the spider (free verbal reports). Based on that, we made the spider slightly smaller, increased the movement speed variation, and included more detailed leg movements. See Fig. 3 for screenshots of the virtual environment. The experiment was presented on two desktop computers, with an Intel Core i9-9820X / Intel Core i7-12700, 16GB RAM, SSD storage and an NVIDIA GeForce RTX 3070.

### Procedure

This study consisted of three parts: an online screening and two laboratory appointments. The general procedure closely resembled the one used by Hollandt et al.^14^. In the online screening, participants provided demographic information and completed the SPQ, FSQ, BDI-II, IUS-12, and STAI-T. If they met the inclusion criteria, they booked a laboratory appointment online. The acquisition and extinction phases took place on two consecutive days to allow for memory consolidation of the learned associations in the acquisition phase. The allocation of colors to the CS+ and CS− conditions was counterbalanced across participants.

**Fig. 4:**
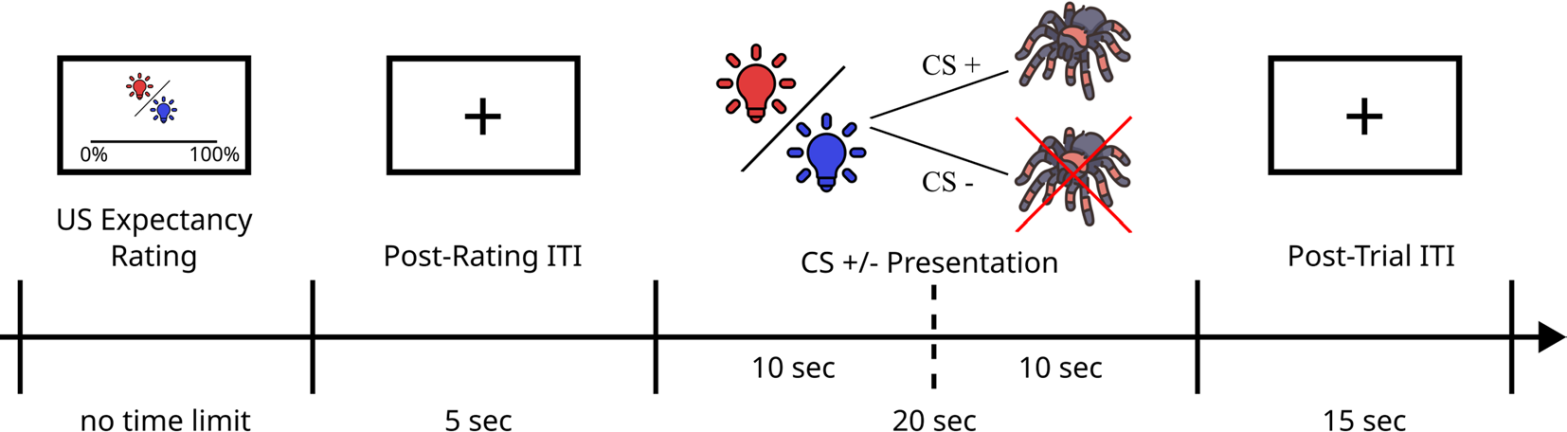
Schematic illustration of the sequence and duration of a single trial. In reinforced CS+ trials, the spider appeared after the first 10 s of CS+ presentation. *CS* conditioned stimulus, *US* unconditioned stimulus, *ITI* intertrial interval.

At the first laboratory appointment, participants first gave written informed consent. The acquisition consisted of four parts: familiarization with the virtual environment, pre-acquisition, acquisition, and stimuli rating. Prior to acquisition, the first-person camera view within the virtual environment was adjusted on the vertical axis to optimally reflect the participant’s real-life height in relation to the virtual table. During familiarization, participants learned how to operate the visual analogue scales (see Fig. 3A) and were allowed to look around in the virtual environment. In pre-acquisition, both CS were presented twice without reinforcement. Participants were instructed which CS would serve as the CS + in the upcoming trials. Both CS were presented 10 times each during acquisition in a randomized order with the constraints that the first CS trial was always a reinforced CS + trial and no CS was presented more than twice in a row. In CS + trials, the CS + was accompanied by a spider in 6 out of 10 trials (60% reinforcement) to increase the uncertainty of the predictive value of the CS+. In reinforced CS + trials, the spider’s movement path was chosen randomly from the six available paths (no path was repeated). Prior to each CS presentation, the upcoming CS was announced and participants provided US expectancy ratings between 0 and 100% on a visual analogue scale (i.e., trial-by-trial US expectancy ratings). See Fig. 4 for an illustration of the detailed time course of a single trial. After acquisition, participants rated the CS+/− and the spider on visual analogue scales for anxiety (0 = *not at all anxiety provoking* to 10 = *extremely anxiety provoking*), disgust (0 = *not at all disgusting* to 10 = *extremely disgusting*), and valence (0 = *very pleasant* to 10 = *very unpleasant*).

The extinction phase on day 2 consisted of two parts: a reacquisition trial and the extinction phase. The reacquisition trial consisted of one reinforced CS + trial to activate the acquired fear memory (the spider always took path 5, Figure S1). In the extinction phase, both CS were presented 20 times without reinforcement. The course of each trial was identical to the acquisition phase (see Fig. 4). At the end of the extinction phase, participants were reimbursed.

### Statistical analyses

Our paradigm included the within-subject factors *CS-Type* (2 levels: CS + vs. CS−) and *Trial* (10 trials per CS in acquisition, 20 trials per CS in extinction). The main outcome of our study was the *US expectancy rating*, while the secondary outcomes were *valence*, *fear* and *disgust ratings* of the CS+/− and the spider.

We conducted all analyses using R^42^, fitting all our models in a Bayesian estimation framework with the R package *brms*^57^ using weakly informative priors.

Generalized linear mixed effects models, in the form of ordered beta regression^58^, were used for the analyses of US expectancies. Ordered beta regression is designed to analyze variables that are bounded between 0 and 1 and also include these bounds as possible values (i.e. ratings such as US expectancies). In our case, we used this model to predict (a) whether the US expectancy rating is zero or continuous, and (b) if it is continuous, a response value between 0 and 1.

We omit ratings of 1 from our model, as we consider this rating to be an outlier in our setting. We also divide all responses by 101 to ensure that they are in the [0, 1] interval for modeling, i.e. we add a small padding for the maximum rating of 100% on the original rating scale. The likelihood of the general model framework for a response *y*_*it*_ from participant *i* in trial *t* is given by:

4 $$f\left( {{y_{it}}|{{{\varvec{\uptheta}}}_{it}},{{{\varvec{\upmu}}}_{it}},\phi } \right)=\left\{ {\begin{array}{*{20}{c}} {\left( {1 - {{{\varvec{\uptheta}}}_{it}}} \right),~~{y_{it}}=0} \\ {{{{\varvec{\uptheta}}}_{it}} \cdot Beta\left( {{{{\varvec{\upmu}}}_{it}},{e^\phi }} \right),~~{y_{it}} \in \left( {0,1} \right),} \end{array}} \right.$$

where ${\text{Beta}}\left( {{{{\varvec{\upmu}}}_{it}},{{\varvec{\uptau}}}} \right)$ is the mean-variance parameterization of the beta distribution. The mean of the beta distribution ${{{\varvec{\upmu}}}_{it}}$ (i.e. the expected rating) and the probability for a continuous rating ${{{\varvec{\uptheta}}}_{it}}$ are further reparametrized in terms of predictors:

5 $$\begin{aligned} {{{\varvec{\uptheta}}}_{it}} &={{\varvec{\upsigma}}}\left( {{{{\varvec{\upzeta}}}_{it}} - {{\varvec{\upgamma}}}} \right), \hfill \\ {{{\varvec{\upmu}}}_{it}} &={{\varvec{\upsigma}}}\left( {{{{\varvec{\upzeta}}}_{it}}} \right), \hfill \\ \end{aligned}$$

where ${{\varvec{\upsigma}}}$ is the logistic function${{\varvec{\upsigma}}}\left( {\text{z}} \right)=\frac{1}{{1+{{\text{e}}^{ - {\text{z}}}}}}$ and ${{{\varvec{\upzeta}}}_{it}}$ is a linear combination of predictors, as in common linear (mixed effects) models. The threshold parameter ${{\varvec{\upgamma}}}$, which acts as an additional intercept for the prediction of zero/non-zero responses, allows us to share the linear prediction term ${{{\varvec{\upzeta}}}_{it}}$ between the discrete and continuous part of the model. For this term ${{{\varvec{\upzeta}}}_{it}}$, we specified mixed effects models with trials as level-one units and individuals as level-two units. The precision parameter $\phi$ determines the variance of the beta distribution and is defined on a log-scale. We estimate one common precision for all responses to reduce model complexity.

We use pseudo-code (similar to the formulas used in the R-package *lme4*^59^) below to describe the models used to operationalize our hypotheses. The left-hand side of these pseudo-code equations corresponds to *y*_*it*_ and the right-hand side to ${{{\varvec{\upzeta}}}_{it}}$, i.e., the part of the model equivalent to linear mixed effects models. The ordered beta framework is therefore omitted in the following model descriptions. As predictors, we use the number of trials (*Trial* in the pseudo code), the *CS-Type*, and IU scores (*IU* in pseudo code) to predict the US expectancy ratings (*ER* in the pseudo code). Intercepts are indicated by *1* and random effects are indicated by parentheses in the pseudo-code. For the manipulation check of valence, fear, and disgust ratings of CS + vs. CS− (H0.2), we used paired t tests. For these t-tests we also provide Cohen’s d as effect sizes. Cohen’s d was calculated using the standard deviation of difference scores (d_z_, see^60^).

## Supplementary Information

Below is the link to the electronic supplementary material.

Supplementary Material 1

## Data Availability

The study’s design, hypotheses, and analysis plan were preregistered on the OSF (https://osf.io/6gjyp). All data, analysis code, and the VR app we developed have been made publicly available at the OSF (https:/osf.io/2nqv4).
