# Supplementary material for: Threat expectancies in a VR fear conditioning paradigm follow non-linear extinction patterns but are not influenced by intolerance of uncertainty
Source: Sci Rep. 2025 Oct 16;15:36125. doi: 10.1038/s41598-025-23629-0 (PMC12532484; doi:10.1038/s41598-025-23629-0)
Supplement: Supplementary file 1 — Supplementary Material 1 [file 41598_2025_23629_MOESM1_ESM.pdf]

Supplement to the article “Investigating threat expectancies via VR fear conditioning: Evidence for non-linear extinction learning and no influence of intolerance of uncertainty“

Markus Grill[1*], Matthias Kloft[2] Steffen Anhäuser[3] & Anke Haberkamp[1]

[1]Department of Psychology and Psychotherapy, University Witten/Herdecke, Germany

[2]Department of Social, Organizational Psychology and Methodology, Philipps Universität Marburg, Germany

[3]Department of Mathematics and Computer Science, Philipps Universität Marburg, Germany

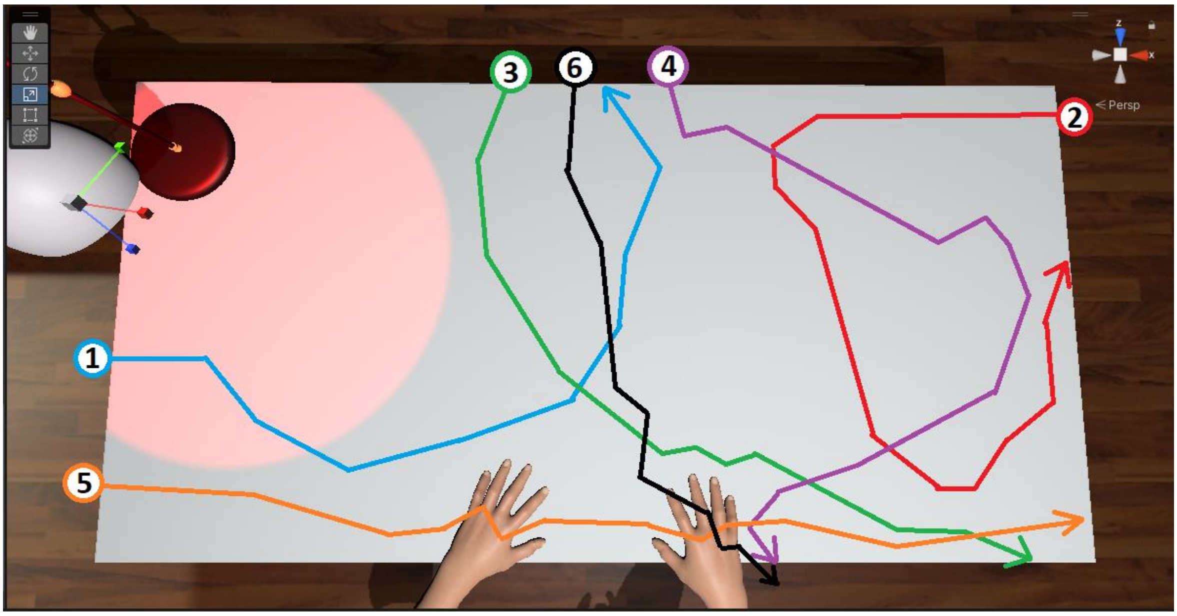

Figure S1. *Illustration of the spiders' possible movement paths in the virtual environment.*

Table S2. *Model parameters for the manipulation check calculated via the ordered beta model.*

Parameter	Est. [95% CI][a]
Fixed effects	
Int.	-0.18 [-0.44; 0.09]
γ	**3.97** [3.61; 4.36]
$b_{CS\text{-}Type}$	**2.56** [2.33; 2.79]
b_{Trial}	-0.02 [-0.06; 0.02]
$b_{CS\text{-}Type \times Trial}$	**0.10** [0.05; 0.14]
Random effects	
ID: Intercept	**0.74** [0.58; 0.93]
ID: *SD*(b_{Trial})	**0.06** [0.02; 0.09]
ID: Cor(Int.,b_{Trial})	**-0.57** [-0.84; -0.17]
Trial: Intercept	**0.13** [0.02; 0.29]
Model fit	Est. [95% CI][b]
R^2 conditional	0.78 [0.77; 0.79]
R^2 marginal	0.73 [0.72; 0.75]

Note. In the random effects section, ID denotes random effects per participant, and Trial denotes the random effect per trial. "R^2 conditional" is based on all effects estimated in the models (i.e. random and fixed effects), while "R^2 marginal" is based only on the estimates for the fixed effects. Model parameters in bold indicate estimates where the 95% CI did not include zero. Cor = correlation.

[a] One-sided lower and upper 95% CIs. [b] Two-sided 95% CIs

Table S3. *Details for different models predicting US expectancies in extinction in response to the CS-, as a control to the CS+ models.*

	Linear Baseline	Ordered Beta Baseline
Parameter	Est. [95% CI]	Est. [95% CI][a]
Fixed effects		
Int.	**0.06** [0.03; 0.09]	**-4.39** [-5.50; -3.44]
γ	n.a.	**2.11** [1.93; 2.30]
b_{Trial}	**0.00** [0; 0]	**-0.29** [-0.46; -0.17]
Random effects		
ID: Intercept	**0.14** [0.11; 0.16]	**3.29** [2.43; 4.44]
ID: $SD(b_{Trial})$	**0.01** [0.01; 0.01]	**0.28** [0.18; 0.43]
ID: Cor(Int.,b_{Trial})	-0.78 [-0.87; -0.67]	**0.35** [-0.11; 0.69]
Trial: Intercept	**0.00** [0.00; 0.00]	**0.05** [0.00; 0.14]
Model fit	Est. [95% CI][b]	Est. [95% CI][b]
R^2 conditional	0.61 [0.59; 0.64]	0.67 [0.87; 0.88]
R^2 marginal	0.01 [-∞; 0.03]	0.01 [0.01, 0.01]

Note. These models included the course of US expectancies during the extinction phase in response to only the CS- as controls to the main CS+ models. The γ parameter is a specific parameter of the ordered beta model and is therefore not present in the linear model. In the random effects section, ID denotes random effects per participant, and Trial denotes the random effect per trial. "R^2 conditional" is based on all effects estimated in the models (i.e. random and fixed effects), while "R^2 marginal" is based only on the estimates for the fixed effects. Model parameters in bold indicate estimates where the 95% CI did not include zero. Cor = correlation.

[a] One-sided lower and upper 95% CIs. [b] Two-sided 95% CIs

Table S4. *Details for the ordered beta models including the STAI-T scores.*

	Ordered Beta<br>STAI-T	Ordered Beta<br>STAI-T + IU
Parameter	Est. [95% CI]	Est. [95% CI][a]
Fixed effects		
Int.	**2.12** [1.78; 2.49]	**2.13** [1.80; 2.47]
γ	**2.36** [2.28; 2.45]	**2.36** [2.28; 2.45]
b_{Trial}	**-0.23** [-0.28; -0.18]	**-0.23** [-0.28; -0.19]
b_{STAI-T}	-0.14 [-0.43; 0.14]	-0.14 [-0.47; 0.20]
b_{IU}	n.a.	**0.01** [-0.33; 0.35]
$b_{Trial \times STAI-T}$	0.01 [-0.04; 0.05]	0.01 [-0.05; 0.06]
$b_{Trial \times IU}$	n.a.	0.01 [-0.05; 0.07]
Random effects		
ID: Intercept	**1.15** [0.95; 1.40]	**1.16** [0.95; 1.40]
ID: *SD*(b_{Trial})	**0.19** [0.16; 0.24]	**0.20** [0.16; 0.24]
ID: Cor(Int.,b_{Trial})	**-0.59** [-0.74; -0.40]	**-0.49** [-0.73; -0.42]
Trial: Intercept	**0.20** [0.13; 0.29]	**0.20** [0.13; 0.30]
Model fit	Est. [95% CI][b]	Est. [95% CI][b]
R^2 conditional	0.88 [0.87; 0.89]	0.88 [0.87; 0.89]
R^2 marginal	0.48 [0.43; 0.53]	0.48 [0.42, 0.52]

Note. These models included the course of US expectancies during the extinction phase in response to only the CS+. In the random effects section, ID denotes random effects per participant, and Trial denotes the random effect per trial. "R^2 conditional" is based on all effects estimated in the models (i.e. random and fixed effects), while "R^2 marginal" is based only on the estimates for the fixed effects. Model parameters in bold indicate estimates where the 95% CI did not include zero. Cor = correlation.

[a] One-sided lower and upper 95% CIs. [b] Two-sided 95% CIs

Upon reviewer request, we provide some general statistics of CS+ / CS- difference scores in the acquisition / extinction phase of our experiment here.

Table S5. *Difference scores for CS+ and CS- in acquisition and general / early / late extinction phases.*

Phase	N	Mean Difference	SD
Acquisition	71	0.66	0.22
Extinction		0.45	0.22
Early Extinction		0.61	0.2
Late Extinction		0.28	0.28

Note. Early extinction: trials 1-10; late extinction: trials 11-20.

Table S6. *Pearson correlations for CS+/CS- difference scores and IU / STAI-T scores across phases.*

	Correlation with difference score			
Phase	IU	I-IU	P-IU	STAI-T
Acquisition	*r* = .085, *p* = .48	*r* = .102, *p* = .40	*r* = .053, *p* = .66	*r* = .035, *p* = .77
Extinction	*r* = -.031, *p* = .80	*r* = -.004, *p* = .98	*r* = -.047, *p* = .70	*r* = -.020, *p* = .87
Early Extinction	*r* = .025, *p* = .84	*r* = -.002, *p* = .99	*r* = .043, *p* = .72	*r* = .017, *p* = .89
Late Extinction	*r* = -.066, *p* = .58	*r* = -.005, *p* = .97	*r* = -.105, *p* = .39	*r* = -.044, *p* = .72

Note. *N* = 71. Early extinction: trials 1-10; late extinction: trials 11-20. (I / P)-IU = (Inhibitory / Prospective) Intolerance of Uncertainty; STAI-T = State-Trait-Anxiety-Inventory – Trait.

Table S7. *Details for the ordered beta model including the SPQ scores.*

	Ordered Beta SPQ
Parameter	Est. [95% CI][a]
Fixed effects	
Int.	1.09 [-0.34; 2.50]
γ	**2.36** [2.28; 2.45]
b_{Trial}	**-0.33** [-0.58; -0.09]
b_{SPQ}	0.04 [-0.03; 0.11]
$b_{Trial×SPQ}$	0.01 [-0.01; 0.02]
Random effects	
ID: Intercept	**1.04** [0.86; 1.26]
ID: *SD*(b_{Trial})	**0.19** [0.16; 0.23]
ID: Cor(Int.,b_{Trial})	**-0.49** [-0.67; -0.28]
Trial: Intercept	**0.20** [0.13; 0.30]
Model fit	Est. [95% CI][b]
R^2 conditional	0.88 [0.87; 0.89]
R^2 marginal	0.50 [0.44, 0.54]

Note. These models included the course of US expectancies during the extinction phase in response to only the CS+. In the random effects section, ID denotes random effects per participant, and Trial denotes the random effect per trial. "R^2 conditional" is based on all effects estimated in the models (i.e. random and fixed effects), while "R^2 marginal" is based only on the estimates for the fixed effects. Model parameters in bold indicate estimates where the 95% CI did not include zero. Cor = correlation. SPQ = Spider Phobia Questionnaire.

[a] One-sided lower and upper 95% CIs.

[b] Two-sided 95% CIs
